# Cellulose Digestion and Metabolism Induced Biocatalytic Transitions in Anaerobic Microbial Ecosystems

**DOI:** 10.3390/metabo4010036

**Published:** 2013-12-31

**Authors:** Akira Yamazawa, Tomohiro Iikura, Yusuke Morioka, Amiu Shino, Yoshiyuki Ogata, Yasuhiro Date, Jun Kikuchi

**Affiliations:** 1 Research Planning and Management Group, Kajima Technical Research Institute, Kajima Corporation, 2-19-1 Tobitakyu, Chofu, Tokyo 182-0036, Japan; E-Mail: akira@kajima.com; 2 Graduate School of Medical Life Science, Yokohama City University, 1-7-29 Suehirocho, Tsurumi-ku, Yokohama, Kanagawa 230-0045, Japan; E-Mails: iikura@tsurumi.yokohama-cu.ac.jp (T.I.); maasii@tsurumi.yokohama-cu.ac.jp (Y.M.); yasuhiro.date@riken.jp (Y.D.); 3 RIKEN Center for Sustainable Resource Science, 1-7-22 Suehirocho, Tsurumi-ku, Yokohama, Kanagawa 230-0045, Japan; E-Mail: amiu.shino@riken.jp (A.S.); 4 Graduate School of Life and Environmental Sciences, Osaka Prefecture University, Osaka 599-8531, Japan; E-Mail: ogata@plant.osakafu-u.ac.jp; 5 Graduate School of Bioagricultural Sciences, Nagoya University, 1 Furo-cho, Chikusa-ku, Nagoya, Aichi 464-0810, Japan; 6 RIKEN Biomass Engineering Program, 2-1 Hirosawa, Wako 351-0198, Japan

**Keywords:** nuclear magnetic resonance (NMR)-based metabolomic approach, heteronuclear correlation (HETCOR), metagenomic analysis, anaerobic ecosystem, carbohydrate-binding module (CBM)

## Abstract

Anaerobic digestion of highly polymerized biomass by microbial communities present in diverse microbial ecosystems is an indispensable metabolic process for biogeochemical cycling in nature and for industrial activities required to maintain a sustainable society. Therefore, the evaluation of the complicated microbial metabolomics presents a significant challenge. We here describe a comprehensive strategy for characterizing the degradation of highly crystallized bacterial cellulose (BC) that is accompanied by metabolite production for identifying the responsible biocatalysts, including microorganisms and their metabolic functions. To this end, we employed two-dimensional solid- and one-dimensional solution-state nuclear magnetic resonance (NMR) profiling combined with a metagenomic approach using stable isotope labeling. The key components of biocatalytic reactions determined using a metagenomic approach were correlated with cellulose degradation and metabolic products. The results indicate that BC degradation was mediated by cellulases that contain carbohydrate-binding modules and that belong to structural type A. The degradation reactions induced the metabolic dynamics of the microbial community and produced organic compounds, such as acetic acid and propionic acid, mainly metabolized by clostridial species. This combinatorial, functional and structural metagenomic approach is useful for the comprehensive characterization of biomass degradation, metabolic dynamics and their key components in diverse ecosystems.

## 1. Introduction

Microbial metabolism of highly polymerized biomass in anaerobic environments, known as anaerobic microbial digestion, is one of the most significant processes on Earth [1]. For example, anaerobic microbial digestion is responsible for biogeochemical cycling in environments, such as oceans and on land, and is used for the processing of industrial organic waste and wastewater [1,2]. This metabolic process is indispensable for maintaining natural environments and conducting industrial activities. It is mediated by complex microbial ecosystems that produce biogas from certain short-chain fatty acids [3]. Therefore, the evaluation of the metabolic dynamics of complex microbial ecosystems, known as microbial metabolomics, involved in anaerobic digestion is a significant challenge for understanding the mechanisms of biogeochemical cycling in the environment and for practical utilization and optimization of industrial processes required to maintain a sustainable society.

To characterize and evaluate biomass degradation and the metabolic dynamics involved in anaerobic digestion that occurs in microbial ecosystems, we focused on cellulose degradation combined with the characterization of the structural heterogeneity of cellulose [4,5]. To address the challenge of this structural heterogeneity, solid-state nuclear magnetic resonance (NMR) spectroscopy provides a powerful tool for characterizing the structure and dynamics of cellulose [6,7,8] and facilitates the characterization of supermolecular structures, such as crystalline and amorphous forms [9,10,11,12,13,14,15]. Further, to evaluate microbial metabolomics, solution-state NMR is a powerful tool for evaluating metabolic dynamics in microbial ecosystems and biological systems present in diverse environments [4,16,17,18,19]. Moreover, we previously developed an analytical method for microbial metabolomics to monitor metabolic dynamics in microbial ecosystems by correlating the relationships between microbial communities and their metabolic activities [20].

In our previous study, the anaerobic microbial digestion of bacterial cellulose (BC) was successfully monitored using solid-, solution- and gas-state NMR spectroscopy with stable isotope labeling [5]. In this anaerobic microbial digestion process of BC, the reactions were initiated from the BC degradation by a microbial community that existed in the ecosystem, producing their enzymes. The BC is a partially crystalline polymer of 1→4-linked β-D-glucose units and exists as crystalline types Iα and Iβ, as well as in an amorphous form. The supermolecular structure of cellulose polymers influences its physical properties and reactivity in synthetic and biological reactions; thus, these complex structures make it difficult to monitor their anaerobic digestion. Therefore, solid-state NMR was used as the most suitable approach to characterize the structural heterogeneity and to monitor the structural variations of BC in the previous study [5]. In addition, the reactions of BC degradation were accompanied with microbial metabolisms, *i.e.*, the metabolic products were produced from the BC by the microbial community. The previous study used an NMR-based metabolomic approach to characterize solution- and gas-state metabolites in combination with the analysis of solid-state BC, because triple-phase NMR spectroscopy is the only method available that analyzes the reactions of solid, liquid and gas phases using a single instrument and characterizes the metabolic dynamics of BC degradation and the production and consumption of short-chain fatty acids and methane. Although this triple-phase NMR approach is a powerful tool for monitoring metabolic conversions of biomass into biogas through short-chain fatty acids by anaerobic digestion, the microbial community and its metabolic functions responsible for the degradation and metabolism of ^13^C-labeled BC (^13^C-BC) were not identified [5].

In the anaerobic digestion process, the key points of the reactions and elements of biocatalytic transitions consist of four prominent types, *i.e.*, the polymeric substrate (BC), the produced metabolites, enzymatic biocatalyst (as players of extracellular degradation) and microbial biocatalyst (as players of intracellular reaction and the production of enzymes and metabolites). As with the above-mentioned and that reported in the previous paper, BC degradation is initially induced by the enzymatic and microbial biocatalyst followed by the structural and conformational changes in BC and metabolites production. For further understanding of the biocatalytic transitions in the anaerobic digestion process, we focused on the characterization of the key players of enzymatic and microbial biocatalyst with time-dependent variations, as well as their relationship between BC degradation and enzymatic biocatalyst variations and produced metabolic profiles and microbial biocatalyst variations.

We here describe the evaluation and characterization of a microbial community and its metabolic functions related to BC degradation in an anaerobic microbial ecosystem. For the evaluation and characterization of the microbial community and its metabolism, we performed metagenomic analysis. The metagenomic analysis enables us to obtain a huge amount of the sequencing data, not only taxonomic information, but also the functional properties compared to conventional microbial community analyses, such as denaturing gradient gel electrophoresis [4,20], and, thus, provides new and deep insight into the relationship between phylogenetic and functional diversity in diverse environments [21,22]. This was accomplished by sequencing genomic material directly obtained from the microbial community. To evaluate microbial metabolic functions, we focused on cellulase, which is one of the key enzymes for the degradation of cellulose, produced by many organisms, including bacteria, Archaea and fungi [23]. Cellulase plays an essential role in BC degradation in anaerobic ecosystems and is a representative member of the glycoside hydrolase family, which hydrolyzes the glycosidic bonds of carbohydrates [24] and typically contains carbohydrate-binding modules (CBMs) [25]. CBMs are classified by their amino acid sequence similarity from which their molecular function can be deduced. Therefore, we considered that it is feasible to determine the microorganisms involved, as well as the conserved domains of certain proteins, CBMs, in particular, which are associated with the process, to evaluate the degradation of supermolecular structure in BC induced by the complex microbial community, including unknown and unculturable microorganisms, using their various catalytic enzymes accompanied by their metabolic products in anaerobic ecosystems.

We here used the E-class web tool [26] for the quantitative analysis of taxonomy and molecular functionality on the basis of metagenomic datasets available in the ECOMICS web toolkit [27], which provides a platform for trans-omics analysis of an environmental sample [28]. E-class enables the biological and functional classification of particular environmental and biological processes through the submission of sequences in a multi-FASTA [29] format and avoids the complicated procedure of uploading and registering the sequences with their detailed metadata. Further, NMR spectral data were processed using FT2DB [28,30], which digitizes one-dimensional (1D) and two-dimensional (2D)-NMR spectra for statistical analysis, as well as using the statistical analysis tool, HetMap, to integrate and display associations between heterogeneous datasets using ECOMICS, as described previously [10]. The variation in data for microbial communities and CBMs classified using E-class on the basis of metagenomic analysis correlated with the biomass degradation profiles measured using solid-state NMR spectroscopy and with metabolic dynamics measured using an NMR-based metabolomic approach according to published studies [4,20]. These correlation analyses revealed the relationships between microbial functions (CBMs) and BC degradation and between the microbial community and its metabolic dynamics (short-chain fatty acid production from the BC biomass).

## 2. Results and Discussion

**Figure 1:**
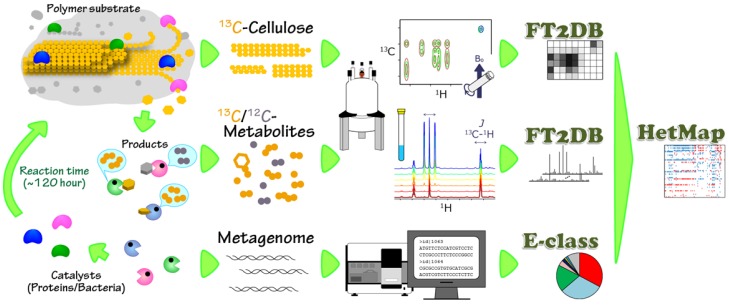
Diagram of the analytical strategy for characterizing bacterial cellulose (BC) degradation in an anaerobic ecosystem. BC degradation and metabolic processes (left cartoon) were characterized using solid- and solution-state NMR spectroscopy with ^13^C-labeled samples (colored in yellow) followed by the digitization of the spectra using FT2DB in the ECOMICS web toolkit. This stable isotope labeling technique promotes the analysis strongly in the mixture state, which could not eliminate the originally existing natural abundance of ^12^C materials (colored in gray), such as fermentation sludge. The biocatalysts, including microorganisms and proteins (carbohydrate-binding modules (CBMs)), characterized by metagenomic analysis, were classified using the E-class web toolkit. The digitized NMR data and categorized metagenomic data were correlated using the HetMap web toolkit in ECOMICS (right scheme).

This study focused on the characterization and elucidation of biocatalytic transitions in the anaerobic digestion process, *i.e.*, the degradation of a supermolecular structure in BC induced by the complex microbial community, including unknown and unculturable microorganisms, using their various catalytic enzymes accompanied by their metabolic products. In commonly-used engineering processes, chemical reactions can be expressed as simpler chemical equations, while the anaerobic digestion process of BC (polymer substrate) is represented by vastly-complicated networks of structural transitions of BC and metabolic (product) dynamics in the microbial community. Because the anaerobic digestion process is intricately involved with a complicated microbial community, including unknown and unculturable microorganisms, accounting for a large portion of the community. To evaluate the biocatalytic transitions, we performed the analysis of a microbial community (catalytic producers) and CBMs (catalytic initiators) responsible for BC degradation accompanied by the evaluation of metabolite production using metagenomic analysis along with an NMR-based metabolomic investigation using the ECOMICS web toolkit (Figure 1). Solid-state ^13^C-^1^H-2D heteronuclear correlation (HETCOR) and solution-state 1D ^1^H-NMR spectra were applied to evaluate BC degradation profiles and metabolomic profiles, respectively. The variation data acquired for the microbial community and CBMs were associated with BC degradation and metabolite production profiles by heterogeneous correlation analysis using HetMap, available from the ECOMICS website (Figure 1).

### 2.1. Characterization of Microbial BC Degradation by Using Solid-State 2D HETCOR NMR

**Figure 2:**
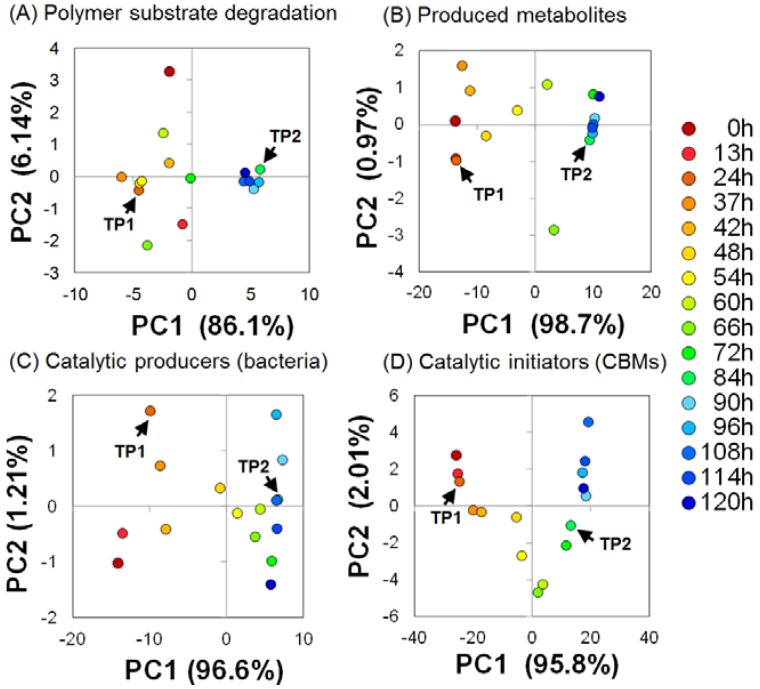
(**A**) Time-course variations in BC degradation (polymer substrate); (**B**) produced metabolites; (**C**) microbial community (catalytic producers); and (**D**) CBMs (catalytic initiator) during the anaerobic digestion process evaluated by principal component analysis (PCA). The microbial community profiles categorized according to “class” are shown in (**C**). Arrows indicate the TP1 (24 h) and TP2 (84 h).

Although BC degradation and metabolic production profiles were characterized previously [5], we further analyzed the ^13^C-BC degradation profiles by solid-state 2D HETCOR NMR spectroscopy, because the binned data of 2D solid-state NMR spectra enabled us to perform high resolution analysis in the loading plots rather than 1D solid-state NMR data, as reported in previous studies [10,11]. Because spin- and dipolar-coupled ^13^C-^1^H cross-peaks were observed in the 2D plane in the time series of HETCOR spectra (Contact Time (CT) = 50 µs), we employed our original 2D binning method, described previously [19,31]. Using 16 time points of numerical data matrices derived from the HETCOR spectra, we calculated principal component analysis (PCA) for monitoring structural changes in ^13^C-BC. The ^13^C-BC degradation profiles evaluated using PCA varied until 84 h after the start of the experiment and then converged on a position on the PCA score plot after 84 h (Figure 2A).

Further, comparative analysis of ^13^C CP-MAS and HETCOR spectra between the initial (0 h), intermediate (60 h) and last points (120 h) revealed that the NMR signals derived from ^13^C-BC were barely detected, but those from lipids and proteins were additionally increased at the last point (Supplementary Figures S1 and S2). For validation of the lipid signal, ^13^C Cross Polarization-Magic Angle Spinning (CP-MAS) NMR measurement was performed at the two conditions of CT = 8 ms and 1 ms. The ^13^C CP-MAS NMR spectra validated that a signal of 29.6 ppm in the ^13^C CP-MAS spectra originated from -(CH_2_)n- in lipids, since it was emphasized, due to the high mobility at CT = 8 ms rather than 1 ms (Supplementary Figure S1). These results suggest that ^13^C-BC was degraded by certain microbes by 84 h after the start of the experiment and is consistent with previously published ^13^C-BC degradation profiles determined using 1D solid-state NMR spectra [5]. Comparison with 50- and 1,000-µs of CT for the same sample suggests that microbial enzymes caused changes in the tertiary structure of ^13^C-BC. Specifically, HETCOR observed at shorter CT (50 µs) did not exhibit significant changes from the initial to intermediate points (zero and 60 h), whereas longer CT (1,000 µs) showed significant changes in the cross-peak intensities of C6-H6/H4, C4-H4/H6 and C1-H6. Note that the corresponding cross-peaks exhibit a characteristic loading plot (Supplementary Figure S3B). This suggests that the tertiary structure of ^13^C-BC, including the packing arrangement of cellulose chains, may change because of the activity of microbial enzymes.

### 2.2. Time-Course Variations in Metabolites, Microbial Community Composition and CBMs

To characterize the transitions of metabolic products derived from ^13^C-BC degradation, we analyzed the solution-state 1D ^1^H-NMR spectra (microbial metabolic profiles) using PCA. The PCA score plots show that the time-course variations in microbial metabolic profiles were observed between 0–84 h (time point 2, TP2) after the start of the experiment and ceased after TP2 (Figure 2B), suggesting that microbial metabolic processes were in progress before TP2. The comparative analysis of ^1^H-NMR spectra between 24 h (time point 1, TP1) and TP2 revealed that the NMR signals derived from ^13^C-labeled metabolites, such as acetic acid, which were possibly derived from ^13^C-BC metabolism by the microbial community, were abundantly produced at TP2 (Supplementary Figure S4). ^13^C-acetic acid levels increased with time and contributed to the separation by PCA from the results of loading plot analysis (Supplementary Figure S3A). This result was consistent with the results of a previous study on metabolic profiles based on ^13^C-NMR spectra [5]. Moreover, the results of solution-state ^1^H-NMR and solid-state HETCOR spectral profiling suggest that a metabolic process generated by the anaerobic microbial ecosystem was caused by ^13^C-BC degradation and was accompanied by the production of metabolites, such as ^13^C-acetic acid and -propionic acid until TP2.

To characterize the microbial community and their metabolic processes that culminated in ^13^C-BC degradation, metagenomic analysis was performed in combination with E-class classification and multivariate statistical analysis. The microbial community (class) and CBM profiles evaluated by PCA varied and shifted from PC1-negative to -positive directions on PCA score plots until approximately TP2 and then varied toward the PC2 direction without any shifts toward the PC1 direction after TP2 (Figure 2C,D). Further, the PCA score plots of the microbial community classified according to phylum (Supplementary Figure S5A), order (Supplementary Figure S5B), family (Supplementary Figure S5C) and genus (Supplementary Figure S5D) showed the same trends in transitions of the microbial community profiles classified according to class. This result indicates that the microbial community degraded ^13^C-BC and produced metabolites until approximately TP2. Moreover, we consider TP2 as a turning point at which microbial activities and functions changed. These trends and transitions are consistent with the results of solution-state ^1^H-NMR and solid-state HETCOR spectral profiling and, therefore, suggest that ^13^C-BC digested by this microbial community and CBMs induced transitions in microbial metabolism to produce acetic acid and propionic acid until approximately TP2.

### 2.3. Key Components of Biocatalytic Transitions in BC Digestion Process

**Figure 3:**
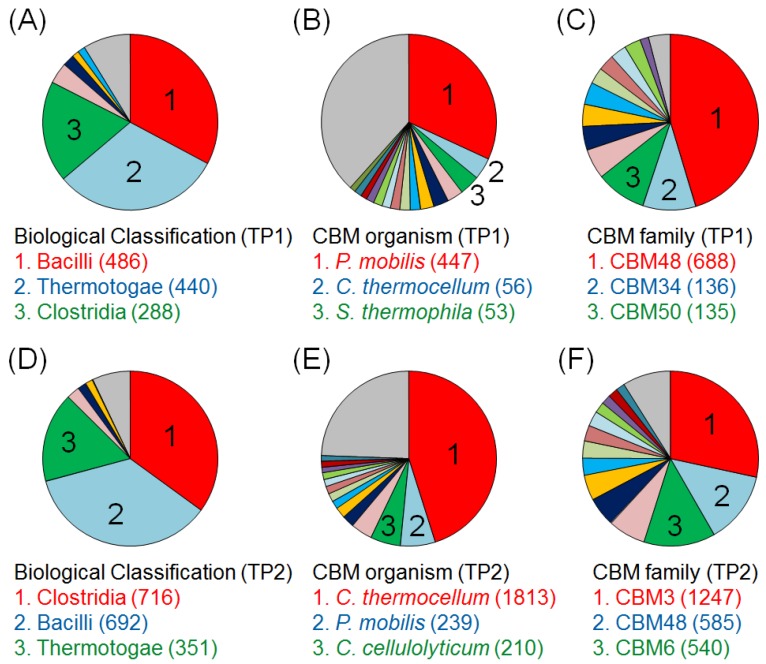
Classification of the microbial community and CBM profiles based on metagenomic analysis data using E-class. The pie charts display taxonomic classification determined from the sequences of 16S rRNA genes (**A**,**D**) or according to the CBMs database (**B**,**E**) and the results of functional classification according to the CBMs database (**C**,**F**) at TP1 (**A**–**C**) and TP2 (**D**–**F**). The top three categories are displayed below the pie charts.

To characterize the key components of biocatalytic transitions (*i.e.*, the responsible microbes and CBMs) in the BC degradation process, we analyzed a dataset that included one million sequence read pairs obtained at TP1 or TP2 using 16S rRNA gene databases and selecting “class” as the taxonomic level. The taxonomic categories with the highest numbers were Bacilli (486 hits), Thermotogae (440), and Clostridia (288) at TP1 (Figure 3A) and Clostridia (716), Bacilli (692) and Thermotogae (351) at TP2 (Figure 3D). These results indicate that the numbers of Clostridia increased during the experiment. Further, we analyzed the sequences using the CBM databases and selected “CBM family,” resulting in CBM48 (688), CBM34 (136) and CBM50 (135) in TP1 (Figure 3C) and CBM3 (1247), CBM48 (585) and CBM6 (540) in TP2 (Figure 3F), indicating an increase in the number of proteins with conserved CBM3 and CBM6 domains. To search for microorganisms classified into CBM3 and CBM6, we analyzed the datasets selected from the analyses of CBM by selecting “Organism” in the “Level” column of the E-class table. These results are listed in Figure 3 and show that bacteria annotated as *C. thermocellum* increased from 56 to 1,813 hits (by a factor of 32; Figure 3B,E). To confirm the reliability of this analysis, we performed a preliminary calculation of the average lengths of individual functional CBM families and found that the lengths of all CBMs and CBM3s were 104 and 81 amino acid residues, respectively, indicating that the numbers of proteins with CBM3 increased significantly from TP1 to TP2. Moreover, sequences annotated as *C. thermocellum* and CBM3 increased from TP1 to TP2, indicating that it is highly possible that bacteria related to *C. thermocellum* (classified as *Clostridium*, the *Clostridiaceae* family, and the Firmicutes phylum) played an important role in the biodegradation of cellulose from TP1 to TP2. However, the numbers of species (rRNA gene sequences) and proteins in public databases are significantly fewer than those of cultured and uncultured microorganisms on the planet. Therefore, if taxonomic and functional information is improved and enriched in the public database, species more closely related to *C. thermocellum* may be detected. Therefore, *C. thermocellum* is tentatively designated as an important bacterium responsible for cellulose biodegradation in the present study. Nevertheless, bacteria closely related to *C. thermocellum* (or clostridial species) likely contributed significantly to ^13^C-BC degradation.

### 2.4. Relationships between Metabolic Dynamics and Biocatalytic Transitions of Microbial Community

**Figure 4:**
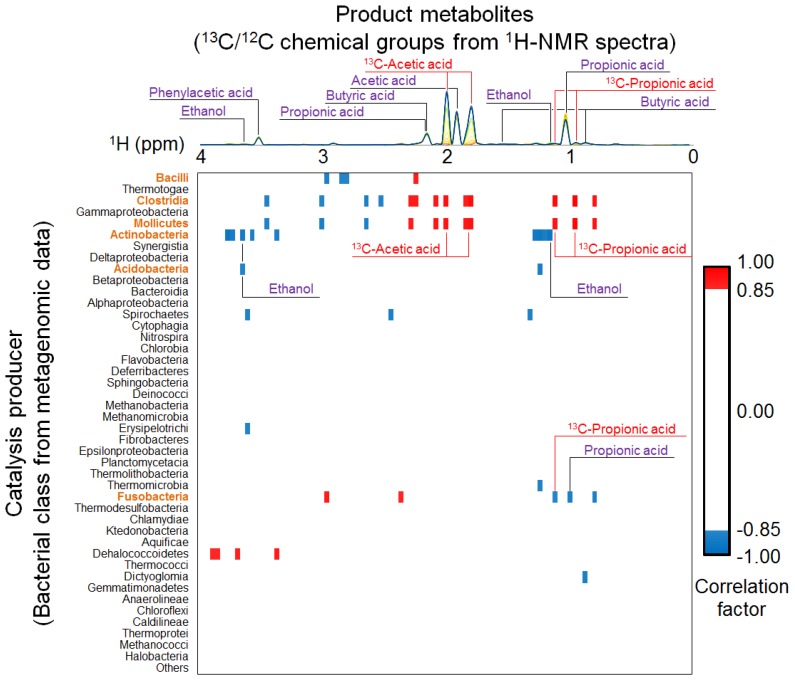
Heterogeneous correlation analysis among taxonomic variations categorized according to “class” from metagenomic data and the solution-state ^1^H-NMR spectra. Note that the metabolite annotations in purple indicate the original ^12^C-derived products, whereas the annotations in red indicate ^13^C-^1^H *J*-coupled signals generated by the metabolism of ^13^C-cellulose. Further, the names of bacterial classes highlighted in orange indicate negative or positive correlations with metabolites. A cutoff score of 0.85 was imposed on the correlation coefficient of the heat map data. Blue and red show negative and positive correlations, respectively.

To evaluate the relationships between microbial community profiles and metabolomic variations in more detail, we performed heterogeneous correlation analysis between taxonomic variations in metagenomic data and solution-state ^1^H-NMR spectra (Figure 4). Bacteria classified as class Clostridia and Mollicutes correlated positively with ^13^C-labeled acetic acid and propionic acid, which were possibly derived from ^13^C-BC. In contrast, bacteria classified as class Fusobacteria correlated negatively with propionic acid synthesis, and those classified as class Actinobacteria and Acidobacteria correlated negatively with ethanol synthesis. These results indicate that ^13^C-acetic acid and -propionic acid were metabolized and synthesized by Clostridia and Mollicutes. Using correlation analysis, we previously demonstrated that the levels of ^13^C-acetic acid and -propionic acid increased as cellulose was degraded, suggesting that the generation of ^13^C-labeled organic acids was caused by the anaerobic digestion of ^13^C-cellulose [5]. Considering our current findings, we suggest that ^13^C-BC was digested by clostridial species and Mollicutes accompanied by the production of acetic acid and propionic acid in the anaerobic microbial ecosystem.

### 2.5. Relationships between BC Digestion and Biocatalytic Transitions of Proteins

**Figure 5:**
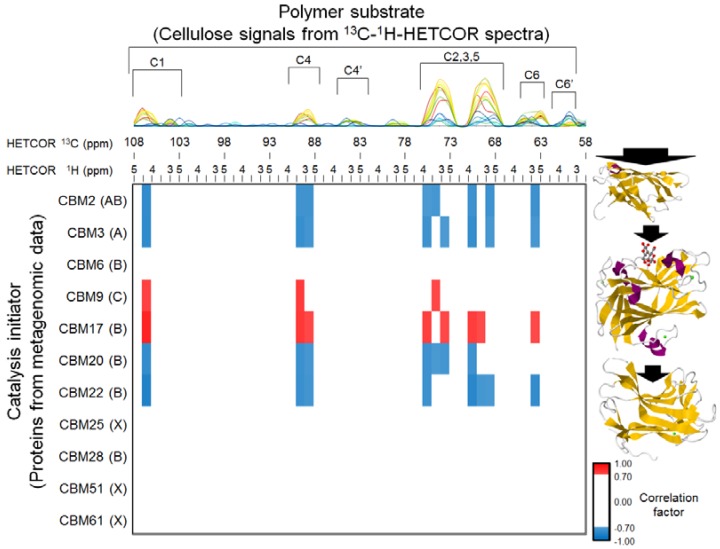
Heterogeneous correlation analysis among functional variations (CBMs) in metagenomic data and structural variation (^13^C-BC) in solid-state heteronuclear correlation (HETCOR) spectra. The 2D HETCOR data were divided into sequential 5-ppm intervals from 58 to 108 ppm for ^13^C chemical shifts and sequential 0.5-ppm intervals from 2.5 to 5 ppm for ^1^H chemical shifts. The integrated ^1^H chemical shifts are arranged from right to left for each ^13^C chemical shift for presentation as 1D data. The three-dimensional structures of the representative structural type A [PDB:1EXG (CBM3)], B [PDB:1H6X (CBM22)], and C [PDB:1I82 (CBM9)] of CBMs are displayed on the right side of the correlation map [32,33,34]. (PDB is Protein Data Bank [35]. Description of structural types after “PDB:” shows structure ID.) Note that carbohydrate-binding surfaces are highlighted with a black arrow on each CBM structure. A cutoff score of 0.7 was imposed on the correlation coefficient of the heat map data. Blue and red show negative and positive correlations, respectively.

To evaluate the relationships between CBM variations and ^13^C-BC degradation profiles in more detail, heterogeneous correlation analysis between functional variations (CBMs) in metagenomic data and the solid-state HETCOR spectra were performed (Figure 5). Proteins categorized as CBM9 and CBM17 correlated positively with the crystalline structure of ^13^C-BC, whereas proteins categorized as CBM2, CBM3, CBM20 and CBM22 correlated negatively. These results indicate that ^13^C-BC degradation was likely related to the proteins categorized as CBM2, CBM3, CBM20 and CBM22, and the bacterial population who carry the proteins categorized as CBM9 and CBM17 were decreased during BC degradation. Some CBM2s and CBM3 are categorized as structural type A, but other CBM2s are categorized as structural type B.

In addition, CBM17, CBM20 and CBM22 are categorized as structural type B, whereas the CBM9 is categorized as structural type C (refer to the representative CBM architecture of structural types A–C, Figure 5) [25]. The type A CBMs slide and diffuse across the surface of cellulose, while the substrate-specific types B and C lock onto their ligands and, thus, direct the enzyme to its target glycosidic bonds [36,37,38]. The type A CBMs (CBM2 and CBM3) bind to the flat, hydrophobic surfaces of cellulose crystals [25,39,40]. Therefore, the proteins categorized as CBM2 and CBM3 likely degraded the crystalline structure of BC, suggesting that they are the major contributors to the microbial digestion of ^13^C-BC. In contrast, proteins categorized as CBM20 and CBM22 that correlated negatively with ^13^C-BC degradation degraded amorphous cellulose and were not likely to degrade crystalline BC. Apparently, the bacteria carrying type A CBMs or the bacteria that increased in number in the ^13^C-BC digestion experiment may synthesize proteins categorized as CBM20 and CBM22. This would explain why CBM20 and CBM22 correlated negatively with the crystalline structure of BC, because CBM classification and quantification are based on sequence-based metagenomic analysis (*i.e.*, an increase or decrease in the detected number of sequences depended on increases or decreases in the bacterial population).

**Figure 6:**
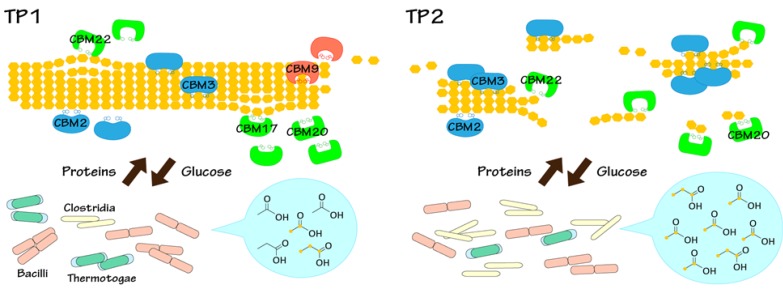
Illustration of the summarized biocatalytic transition for BC degradation in the anaerobic digestion ecosystem. The difference in the biocatalytic transition at TP1 and TP2 is summarized in this figure. Chains of yellow hexagons mean ^13^C-BC as the polymer substrate. ^13^C-labeled products metabolized from ^13^C-BC show up as yellow nodes in the structural formula.

The results of HETCOR and the metabolomic analysis data show that the relative peak intensities of cellulose (approximately 55–110 ppm) [41,42,43,44,45] decreased with time, while the levels of metabolites, such as ^13^C-acetic acid and -propionic acid increased (Supplementary Figures S1, S2 and S4). By integrating the taxonomic and functional classification using E-class with solid-state HETCOR measurements and NMR-based metabolomic profiles, we revealed that ^13^C-BC was degraded mainly by Clostridia that produced cellulases classified as CBM2 and CBM3, accompanied by the conversion of ^13^C-BC to ^13^C-acetic acid and -propionic acid (Figure 6). On the other hand, the bacterial population who carry the proteins categorized as CBM9 and CBM17 were decreased during BC degradation. Taken together, this study was able to characterize the structural and conformational changes in the BC degradation process, to identify the key microbial players and enzymes of the degradation process, to track and monitor the metabolic dynamics in the microbial community using stable-isotope labeling, to link the microbial community and activity with structural and conformational changes of BC and to produce metabolites from the BC.

Therefore, these findings indicate that monitoring biomass degradation and metabolic profiles in microbial ecosystems using solid- and solution-state NMR spectroscopy with isotope labeling technology provides a powerful tool for evaluating molecular and metabolic dynamics. By combining these techniques with metagenomic approaches based on next-generation sequencing, the approach described here may be used for functional and structural metagenomic analysis. It should also be capable of deciphering complicated metabolic networks and dynamics induced by biomass degradation and revealing key microbes and how they function in this process, particularly when metagenomic datasets become more robust as metagenomic technologies improve. This will lead to a better understanding of the organisms involved and their mechanisms of action that contribute to environmental and biological processes.

## 3. Experimental

### 3.1. General

The materials and samples, including ^13^C-BC produced by *Gluconacetobacter xylinus* used in this study, were prepared as described previously [5]. Briefly, powdered ^13^C-BC was incubated in stirred tank reactors at a constant temperature of 55 °C for 120 h under anaerobic condition with thermophilic anaerobic digestion sludge. Solid cellulosic samples and solutions of metabolite mixtures, as well as bacterial DNAs were sampled at 16 time points from 0 to 120 h during anaerobic fermentation. Metagenomic analyses to evaluate the microbial community and CBMs were performed using an Illumina GAIIx (Illumina Inc., San Diego, CA, USA). The variations in metagenomic profiles were analyzed using multivariate statistical analysis and correlated with BC degradation profiles determined from solid-state HETCOR spectra recorded on a Bruker DRX-800 spectrometer and microbial metabolomic profiles determined from solution-state ^1^H-NMR spectra recorded on a Bruker DRX-700 spectrometer using the ECOMICS web toolkit.

### 3.2. NMR Spectroscopy

Solid-state 2D HETCOR spectra were recorded using an DRX-800 spectrometer (Bruker-BioSpin, Billerica, MA, USA) with a Bruker 4-mm Magic Angle Spinning (MAS) triple resonance probe, as described previously [5]. The MAS frequency, the contact time and the recycle delay for HETCOR was fixed at 12 kHz, set to 50 and 1,000 µs and set to 1.2 s, respectively. Note that 16 time points acquired with CT = 50 µs were used for the PCA calculation as follows. For the 2D HETCOR spectra, the data were processed using the NMRPipe software [46] and reduced by subdividing the spectra into sequential 5-ppm regions between ^13^C chemical shifts of 58–108 ppm and sequential 0.5-ppm regions between ^1^H chemical shifts of 2.5–5 ppm using FT2DB software [30] in the ECOMICS web toolkit. Solid-state 1D CP-MAS spectra were recorded on a Bruker DRX-800 spectrometer with CT = 8 ms and 1 ms, as described previously [5]. Solution-state 1D ^1^H-NMR spectra were recorded on a Bruker AV700 spectrometer, as described previously [5].

### 3.3. DNA Extraction and Metagenomic Dataset Preparation

Genomic DNAs were extracted from the samples according to a previous report [20]. In brief, the samples were centrifuged and separated into precipitates and supernatants. The precipitates were dried under vacuum and milled in a solution of 10% sodium dodecyl sulfate to crush the microbial cells. The genomic DNAs were then extracted from the crushed samples. We outsourced the preparation of the metagenomic dataset to RIKEN GENESIS Co. Ltd. (Yokohama, Japan). Sequence libraries comprising microbial sequences (averaging 180 bp) were generated from the DNA samples, and metagenomic datasets (valid paired-end sequences with 75 and 72 bases) were acquired from the libraries using the Illumina GAIIx. We obtained sequence datasets of the individual samples, including approximately 1 million paired-end sequences, which are available on the E-class website [26].

### 3.4. Classification of the Microbial Community and CBMs Using the E-Class Web Tool

For taxonomic and functional categorization using 16S and 18S rRNA genes and the CBM domain, we used the E-class web tool available on the ECOMICS website [27,28]. For the analysis of the microbial community and CBMs involved in BC degradation, paired-end sequences were employed without assembly into contigs using the absolute number of sequences for the taxonomic and functional classification based on 16S rRNA genes and CBM in the E-class databases. The classification and categorization of the metagenomic dataset was performed by drawing pie charts simultaneously with the E-class web tool.

### 3.5. Statistical Analysis

For the multivariate statistical analysis, we used the recycle delay of 2 s in the NMR measurements to focus on numerical analysis of the variations of “relative” abundance, especially mobile components, based on the intensities of NMR spectra, as described in a previous report [47], not the quantification of the “absolute” abundance. Solution-state NMR data were reduced by subdividing the spectra and were processed and normalized for statistical analysis, as described previously [5]. PCA of the binned data was performed using “R” software according to a previous study [48]. A 2D heterogeneous correlation map was calculated as an asymmetric matrix between the variation data for CBM categorized using E-class and biodegradation profiles of BC based on HETCOR data and between microbial community profiles based on metagenomic data and microbial metabolomic profiles obtained from solution-state ^1^H-NMR data analyzed using Spearman’s rank correlation coefficient on HetMap [49] in the ECOMICS web toolkit [4,10,28].

## 4. Conclusions

We conducted a comprehensive analysis of the degradation of ^13^C-BC by a microbial community. This analysis characterized the metabolites produced, the CBMs of the cellulases involved and the bacteria that were the most likely sources of these enzymes, determined using solid- and solution-state NMR profiling in combination with a metagenomic approach. These analyses indicated that ^13^C-BC was degraded by CBM2 and CBM3 cellulases. Clostridial species, which were highly abundant, were the most likely sources of these cellulases and produced metabolic compounds, such as ^13^C-acetic acid and -propionic acid from ^13^C-BC. The NMR-based metabolomic approach in combination with biomass profiling using solid-state NMR and metagenomic analysis described here is useful for the comprehensive characterization of biomass degradation, metabolic dynamics and the responsible microbes present in various environmental ecosystems. This methodology promises to be useful for deciphering complicated metabolic processes, networks and interactions within microbial communities.

## Supplementary Files

Supplementary File 1 Supplementary File (PDF, 322 KB)
