# Supplementary material for: Cellulose Digestion and Metabolism Induced Biocatalytic Transitions in Anaerobic Microbial Ecosystems
Source: Metabolites. 2013 Dec 31;4(1):36–52. doi: 10.3390/metabo4010036 (PMC4018678; doi:10.3390/metabo4010036)

Supplementary File

Figure S1. Solid-state ^{13}C Cross Polarization-Magic Angle Spinning (CP-MAS) spectra at 0 h (red), 60 h (green) and 120 h (blue) for the conditions of Contact time (CP) = 8 ms (lower) and 1 ms (upper). Peaks highlighted by a red asterisk are the methyl of sodium acetate added as an internal standard. Proteins "bb" and "sc" mean "backborn" and "side-chain", respectively.

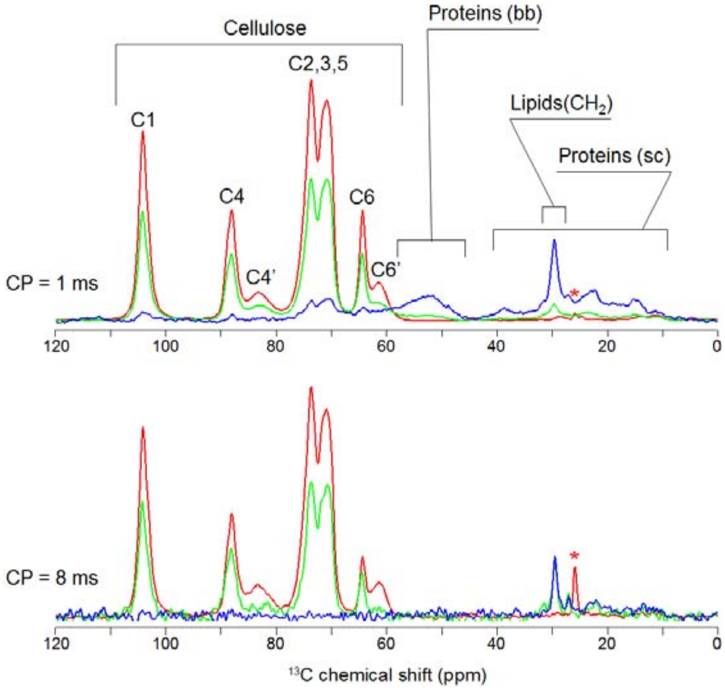

Figure S2. Solid-state 2D HETCOR spectra measured using CP periods of 50 µs (left) and 1,000 µs (right) sampled at the initial (0 h: upper), intermediate (60 h: middle) and last points (120 h: lower) of the experiments. Peaks highlighted by a red asterisk are the methyl of sodium acetate added as an internal standard.

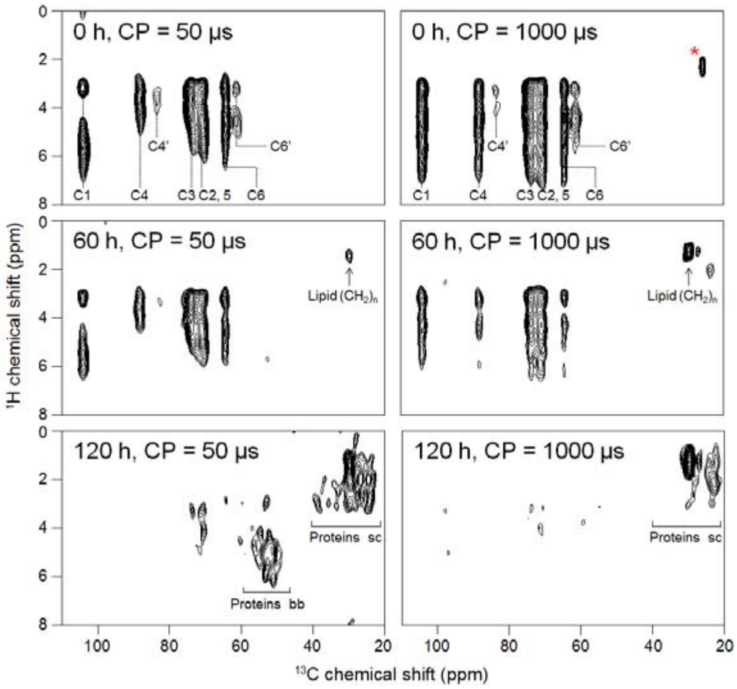

Figure S3. (**A**) Loading plots of solution-state ^{1}H-NMR spectra shown in Figure 2B; and (**B**) solid-state 2D HETCOR spectra (CP = 50 μs) shown in Figure 2A.

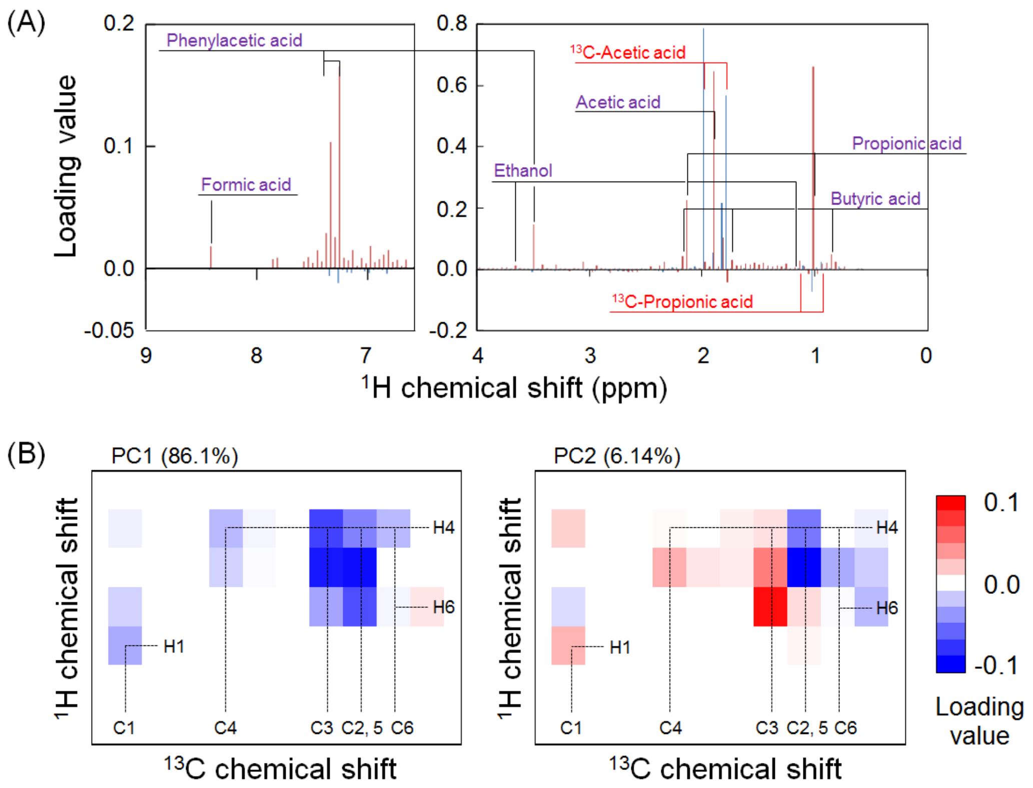

Figure S4. Stacked plots of solution-state ^{1}H-NMR spectra at TP1 (brown) and TP2 (green). Note that the metabolite annotations in purple indicate original ^{12}C-derived products, whereas the annotations in red indicate ^{13}C-^{1}H J-coupled signals metabolized from ^{13}C-cellulose.

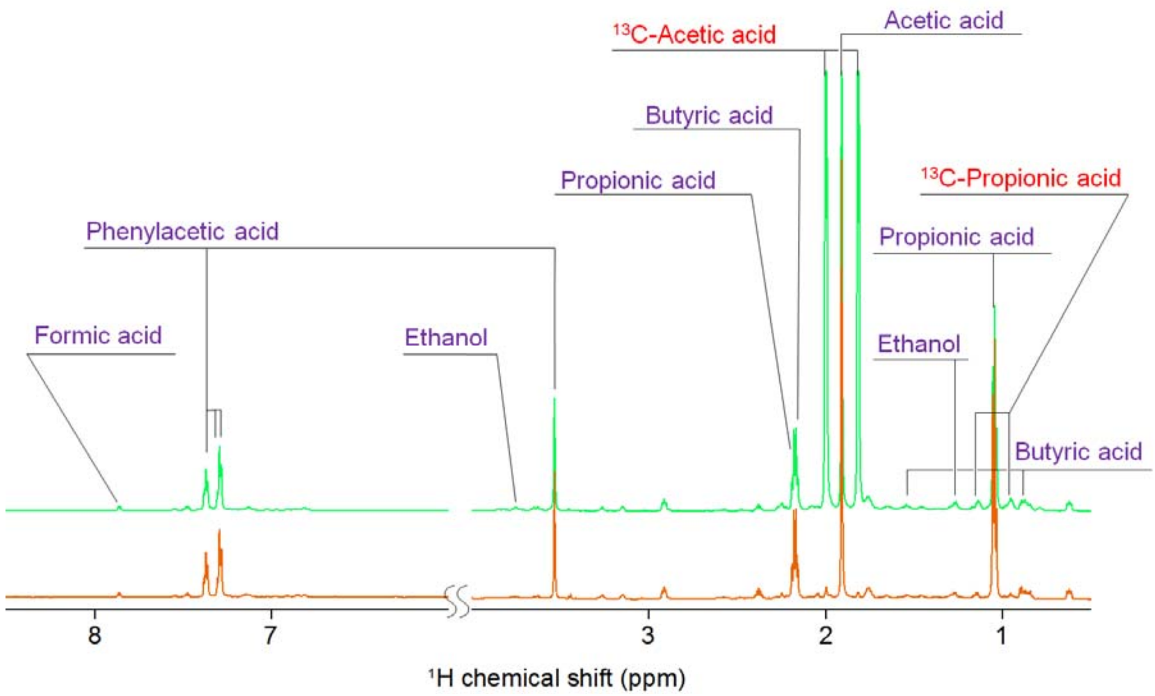

Figure S5. Time-course variations in microbial community profiles classified according to (**A**) phylum; (**B**) order; (**C**) family; and (**D**) genus during the anaerobic digestion process evaluated by PCA. Arrows indicate the TP1 (24 h) and TP2 (84 h).

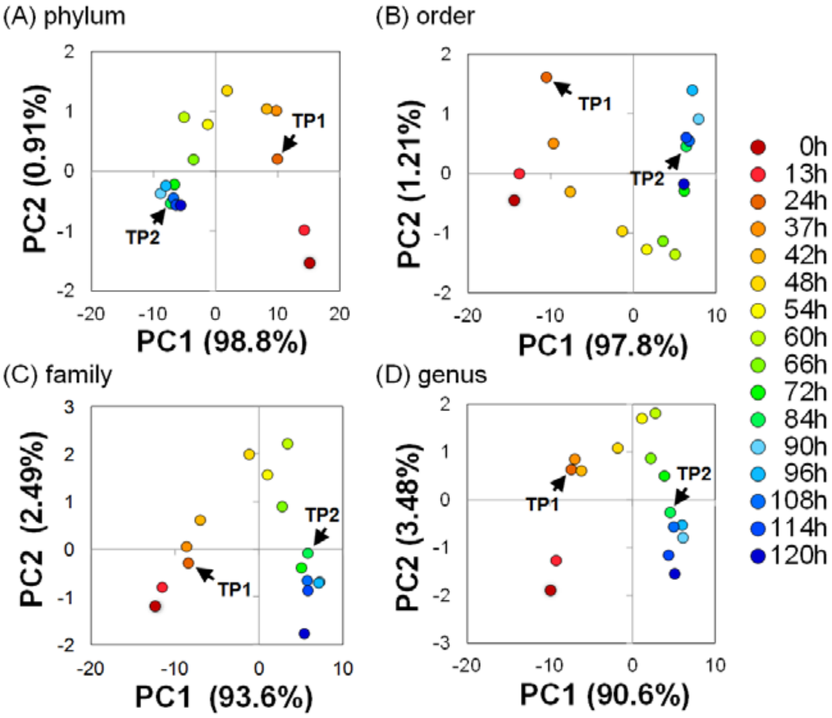

Supplement: Supplementary File 1 — Supplementary File (PDF, 322 KB) [file metabolites-04-00036-s001.pdf]
